# Biomarkers in acute kidney injury: On the cusp of a new era?

**DOI:** 10.1172/JCI171431

**Published:** 2023-07-03

**Authors:** Mark Canney, Edward G. Clark, Swapnil Hiremath

**Affiliations:** 1 Division of Nephrology, Department of Medicine, University of Ottawa and the Ottawa Hospital, Ottawa, Ontario, Canada.; 2 Clinical Epidemiology Program, Ottawa Hospital Research Institute, Ottawa, Ontario, Canada.

## Abstract

The field of nephrology has been slow in moving beyond the utilization of creatinine as an indicator for chronic kidney disease and acute kidney injury (AKI). Early diagnosis and establishment of etiology, in particular, are important for treatment of AKI. In the setting of hospital-acquired AKI, tubular injury is more common, but acute interstitial nephritis (AIN) has a more treatable etiology. However, it is likely that AIN is under- or misdiagnosed due to current strategies that largely rely on clinical gestalt. In this issue of the *JCI*, Moledina and colleagues made an elegant case for the chemokine called C-X-C motif ligand 9 (CXCL9) as a biomarker of AIN. The authors used urine proteomics and tissue transcriptomics in patients with and without AIN to identify CXCL9 as a promising, noninvasive, diagnostic biomarker of AIN. These results have clinical implications that should catalyze future research and clinical trials in this space.

## Biomarkers in kidney disease

The search for a better biomarker of acute kidney injury (AKI) to replace serum creatinine has been long and elusive (1). The field of cardiology has utilized various tests through the years to indicate myocardial injury, progressing from creatine kinase to creatine kinase–myocardial band, to troponin, to troponin subtypes, to highly sensitive troponin subtypes. This evolution has improved diagnostics, risk-stratification, acute care processes, and prognostication for patients with suspected myocardial injury. Meanwhile, renal medicine has remained stuck in the creatinine first gear. The cynics may point out that the heart is a sophisticated, but, for all purposes, glorified muscle tissue, whereas the kidney is a much more elegant organ with filtering, secretory, synthetic, and endocrine functions to maintain homeostasis and much more. The kidney also has many more cell types than the heart, which complicates the matter of utilizing a simple injury biomarker like troponin**.** Nephrology research in the last few decades has, indeed, revealed a rich tapestry of candidate biomarkers, traversing cellular injury, cell cycle arrest, and repair, all helping to provide a more specific diagnosis beyond the identification of AKI (1, 2).

## Diagnostic dilemmas in AKI

This need to pinpoint the differential diagnosis also applies for hospital-acquired AKI, despite the range of diagnoses being narrower than that for AKI discovered in other settings. The pretest likelihood for acute tubular injury is typically high in the context of most hospital-acquired AKI following an intercurrent illness such as sepsis, a major adverse cardiovascular event, or complications after surgery. Despite this probability for tubular injury, the stakes for an accurate diagnosis are higher so as not to miss a potentially treatable condition (3). In particular, clinicians do not want to miss AKI due to acute tubulointerstitial nephritis (AIN), because, unlike nonspecific tubular injury, clinicians can usually ameliorate AIN by removing the offending medication. This otherwise well-intentioned intervention has substantial implications for patients. It is not a trivial decision to empirically stop medications such as antimicrobial agents during severe infection or chemotherapy in a patient with cancer. The other intervention available involves prescribing corticosteroids that dampen the inflammation. Needless to say, high-dose corticosteroids have myriad unwanted effects that can make a bad situation worse in a sick patient. A kidney biopsy may clarify the diagnosis of AIN and help to establish the net benefit of these actions, but a biopsy carries above-average risk in an unwell patient and could be contraindicated, for example, due to the need for antiplatelet or anticoagulant therapy. In the absence of a biopsy, clinicians are left shooting in the dark with highly inaccurate tools, such as a blood eosinophil count and urine microscopy findings, to guide them (4–6). Ultimately, a diagnosis of AIN is often based on clinical gestalt. It’s also possible that clinicians miss a portion of AIN cases due to a lack of clinical suspicion for this condition. Compared with other conditions of the kidney, most notably the rapidly evolving understanding of glomerular disease mechanisms and related therapeutic targets, there has been little innovation in the approach to AIN. Notably, as a testament to the difficulties in diagnoses and navigating the therapeutic interventions, there have been zero trials to date conducted in the AIN setting, and marked uncertainties persist even in the role for corticosteroid use (7).

## CXCL9 as a biomarker for AIN

In this issue of the *JCI*, Moledina and colleagues propose that a chemokine called C-X-C motif ligand 9 (CXCL9) can be used as a biomarker to reliably identify AIN in patients with AKI (8). CXCL9 mediates most of its biological function through binding to CXCR3, a seven-transmembrane-domain receptor coupled to G proteins (9). CXCL9 primarily attracts activated T lymphocytes that express high levels of CXCR3. CXCR3 expression is induced primarily by the Th1-associated cytokine IFN-γ and correlates with tissue infiltration of T lymphocytes in a number of Th1-associated diseases, suggesting that CXCL9 plays an important role in the regulation of effector cell recruitment to sites of inflammation. With respect to the kidney, CXCL9 has previously been implicated in inflammatory states such as kidney allograft rejection, and its expression appears to be restricted to the tubulointerstitial compartment, making it an attractive prospect for the study of AIN (10).

Moledina and colleagues carried out a series of experiments to test their hypothesis (8). First, in a sample of 88 participants — 31 with biopsy-confirmed AIN — they conducted urine proteomic analysis of 180 candidate proteins and demonstrated that CXCL9 had the strongest association with AIN, being 7.6-fold higher in participants with AIN compared with individuals in the control group. Second, they measured CXCL9 using a modified sandwich immunoassay in urine samples from 204 consecutive patients who underwent a kidney biopsy for assessment of AKI — 31 patients with AIN. CXCL9 levels were 5.5-fold higher in patients with AIN compared with other causes of AKI and 8-fold higher compared with patients with acute tubular injury. Third, they generated a logistic regression model to evaluate the association between CXCL9 and AIN diagnosis. Compared with the lowest quartile, the highest quartile of CXCL9 was associated with 6-fold higher odds of AIN (odds ratio 6.0, 95% CI 1.8–19.9), and this estimate was not attenuated after adjusting for readily available laboratory tests that were included in a previously validated diagnostic model for AIN (5). The addition of CXCL9 to this model led to an improvement in discrimination, reflected by the AUC measure, which increased to 0.82 from 0.74. CXCL9 also out performed two previously identified AIN biomarkers: TNF-α and IL-9 (8, 11, 12). Importantly, the association between CXCL9 and AIN was consistent in two external patient cohorts. Fourth, the investigators compared tissue expression of CXCL9 in patients with (*n* = 59) and without (*n* = 52) AIN. Tissue mRNA expression of CXCL9 was higher in biopsy tissue from patients with AIN compared with other kidney conditions and individuals in the control group. Finally, the authors explored combinations of all 16 biomarkers that were measured in the cohorts to identify the optimal set of biomarkers for AIN diagnosis. For this purpose, the investigators employed least absolute shrinkage and selection operator (LASSO), a variable selection technique that guards against model over-fitting and can handle correlated variables. The combination of CXCL9, TNF-α, and IL-9 were selected in more than 75% of model iterations and culminated in excellent diagnostic accuracy with an AUC of 0.89 in the discovery cohort and 0.87 in the external validation cohort (8).

## Clinical and research implications

**Figure 1:**
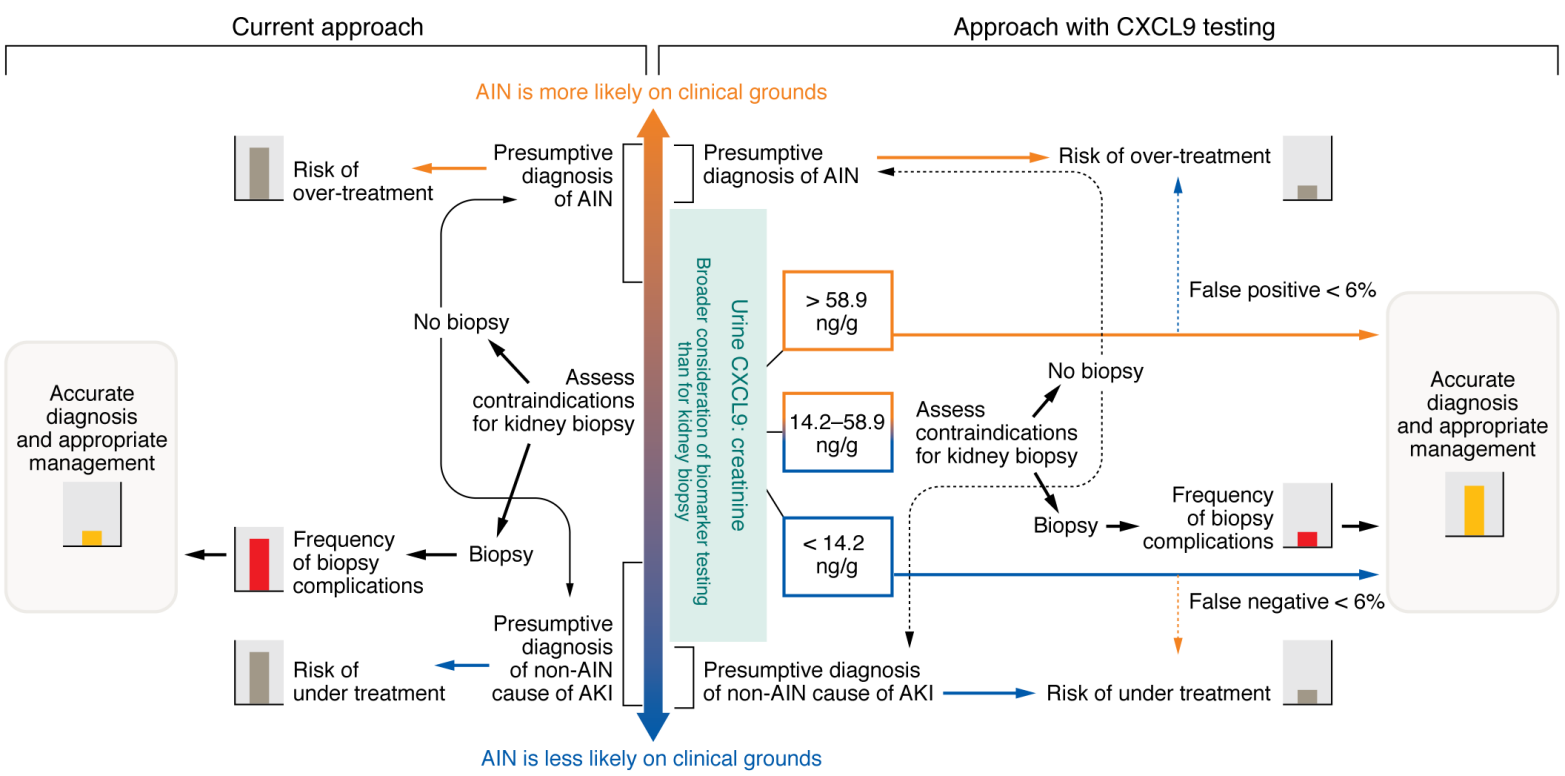
A framework that incorporates testing for urinary CXCL9 may improve the accuracy of diagnosis and management of AKI. Clinicians rely on kidney biopsies under the current approach to diagnose AIN. However, if the biopsy is contraindicated, the risk for over or under treatment remains high. If the biopsy is performed, accuracy of diagnosis is accompanied by a higher frequency of biopsy-related complications. Use of biomarker testing beyond biopsy may improve the accuracy of diagnosis while mitigating risk for complications. Analysis of urinary CXCL9 alongside creatinine could establish a diagnostic strategy that decreases the risk of false positive and false negative diagnosis. Moledina et al. (8) established cut points for urinary CXCL9-to-creatinine ratios, in which values above 58.9 ng/g ruled in AIN, while those below 14.2 ng/g suggested another cause for AKI. This approach may indicate causes of AKI at early time points when prevention of further kidney damage is possible.

The Moledina, et al. study findings are exciting because they provide a road map of where diagnostics can get to for this common, yet poorly identified and treated, cause of kidney damage (8). The need for a different approach can be readily identified from the fact that clinicians’ gestalt for diagnosing AIN was almost tantamount to tossing a coin (AUC of 0.57) (8). CXCL9 alone out performed not only the clinician’s prebiopsy suspicion, but also an existing diagnostic model and other candidate biomarkers both in the discovery and external validation cohorts. Intriguingly, the combination of CXCL9, TNF-α, and IL-9 had the best diagnostic accuracy (8), and a multimarker approach is likely to become the favored approach in future studies (Figure 1). This multimarker strategy also has the potential to provide a point-of-care, noninvasive diagnostic test for AIN, which would be a huge advance compared with the current standard of care. There is a long way to go before achieving this goal, however, and several uncertainties remain. Like other pathological kidney diagnoses, AIN is not a single entity, and it is driven by many different underlying processes, including hypersensitivity reactions to drugs, autoimmune conditions, and specific mechanisms of injury such as that caused by immune checkpoint inhibitors for the treatment of certain cancers. The diagnostic capacity of biomarkers such as CXCL9, alone or in combination, will need to be tested in larger samples of patients with different phenotypes of AIN to refine our understanding of its pathogenesis. Although Moledina and authors noted a cross sectional association between the value of CXCL9 and pathological features of disease severity on kidney biopsy, a dose-response relationship was not established and the kinetics of CXCL9 could vary depending on the evolution of kidney injury and affect interpretation (8). There is an inherent trade off with the use of any biomarker depending on the clinical situation and the gravity of making an error. Choosing a high threshold value of CXCL9 for AIN diagnosis would be useful if the goal is to have a rule-in test to provide justification to start empiric therapy with corticosteroids, whereas adopting a lower threshold might be preferable if the index of suspicion for AIN is low and the presence of a determined normal range test result provides a high-negative predictive value. There is a large range of CXCL9 values between these two thresholds, which may not advance the diagnostic algorithm very far, especially if a kidney biopsy is deemed hazardous.

In this thoughtfully designed and well-executed study, Moledina and colleagues provide a compelling argument for CXCL9 as a promising, noninvasive diagnostic biomarker in distinguishing AIN from other causes of AKI (8). Alongside the potential for CXCL9 and other biomarkers to improve care pathways, inform risk, and/or benefit discussions with patients experiencing AKI, this biomarker could also potentially allow a more enriched population for future clinical trials (13). Additionally, the translational approach employed in this study also generates hypotheses regarding key players in the pathogenesis of AIN that should be the catalyst for future research questions in this space. While the holy grail of a urine dipstick test for AIN may seem aspirational, it is certainly time to move beyond current rudimentary clinical tools and embrace innovative methods for the evaluation and earlier detection of potentially reversible causes of AKI.

## Version 1 07/03/2023

Electronic publication
